# Comment on “Effect of Pumpkin Seed Oil on Hair Growth in Men with Androgenetic Alopecia: A Randomized, Double-Blind, Placebo-Controlled Trial”

**DOI:** 10.1155/2015/271474

**Published:** 2015-03-31

**Authors:** Hitesh Verma

**Affiliations:** Overseas R&D Centre, Overseas HealthCare Pvt. Ltd., Phillaur, Punjab 144410, India

Androgenetic alopecia (AA) or common baldness is one such condition which affects almost every male at some stage of his life, depending upon his genetic susceptibility. Though AA is not associated with any health related issue but it has a lot of psychological effect on men especially on his self-confidence [1]. Currently practiced therapy include finasteride (1 mg), dutasteride (2.5 mg), and minoxidil lotion (2% and 5%), but they have their own side effects (e.g., effect on sexual health, scalp itching, and scaling) [2, 3]. This makes the consumer to shift towards natural therapies. Currently the most practiced natural therapy include saw palmetto (both oral and lotion) [4, 5], but its efficacy is questioned from time to time. Cho et al., in 2014 [3], represent in their research that pumpkin seed oil (PSO) can open a new era in AA treatment. Though the primary results shown by them are quite promising, there are many questions left unanswered. Research data seems to be incomplete; since no data was reported related to the effect of PSO on frontal AA, only pictorial representation of vertex AA is reported in the study. It is important to note that the volunteers of Norwood-Hamilton types II, III, III vertex, IV, and V were enrolled in study and investigator analysis for frontal AA was also done but results were not mentioned [3]. Frontal AA is required to be discussed because of two reasons; firstly, AA can be reasonably treated if treatment will be availed during the early stage of progression [6]; therefore, effect of PSO on Norwood-Hamilton types II and III should be discussed in future studies. Secondly, in one of the studies comparing botanical treatment of AA (saw palmetto) and finasteride, it was observed that botanical treatment was not effective in treating frontal AA [7]; therefore, to ascertain the possibility of ineffectiveness or effectiveness of PSO on frontal AA, it is suggested to Cho et al. to avail data relating the effect of PSO on frontal AA as it will clear the picture to some extent, though future studies are also desirable. Another thing which is important and is a matter of future research is appropriate duration of the study. Study duration should be at least 1 year because there is marked effect of seasonal variation on hormonal levels in males (being peaked during summer and being the least during autumn) which in turn affect the anagen phase of hair growth [6]. I highly appreciated the results of Cho et al. and look forward for the clinical research ascertaining the effect of PSO on frontal AA and variability in its results during long term usage.
